# Engineering of the genome editing protein Cas9 to slide along DNA

**DOI:** 10.1038/s41598-021-93685-9

**Published:** 2021-07-08

**Authors:** Trishit Banerjee, Hiroto Takahashi, Dwiky Rendra Graha Subekti, Kiyoto Kamagata

**Affiliations:** 1 Institute of Multidisciplinary Research for Advanced Materials, Tohoku University, Katahira 2-1-1, Aoba-ku, Sendai, 980-8577 Japan; 2 Department of Chemistry, Graduate School of Science, Tohoku University, Katahira 2-1-1, Aoba-ku, Sendai, 980-8577 Japan

**Keywords:** Single-molecule biophysics, Protein design, DNA-binding proteins

## Abstract

The genome editing protein Cas9 faces engineering challenges in improving off–target DNA cleavage and low editing efficiency. In this study, we aimed to engineer Cas9 to be able to slide along DNA, which might facilitate genome editing and reduce off-target cleavage. We used two approaches to achieve this: reducing the sliding friction along DNA by removing the interactions of Cas9 residues with DNA and facilitating sliding by introducing the sliding-promoting tail of Nhp6A. Seven engineered mutants of Cas9 were prepared, and their performance was tested using single-molecule fluorescence microscopy. Comparison of the mutations enabled the identification of key residues of Cas9 to enhance the sliding along DNA in the presence and absence of single guide RNA (sgRNA). The attachment of the tail to Cas9 mutants enhanced sliding along DNA, particularly in the presence of sgRNA. Together, using the proposed approaches, the sliding ability of Cas9 was improved up to eightfold in the presence of sgRNA. A sliding model of Cas9 and its engineering action are discussed herein.

## Introduction

Clustered regularly interspaced short palindromic repeats (CRISPR)-associated protein Cas9 has been widely used for genome editing. Cas9 from *Streptococcus pyogenes* is an RNA-guided nuclease that originates in an adaptive immune system to fight off foreign genetic elements. Cas9 is composed of an α-helical recognition (REC) lobe and a nuclease (NUC) lobe, including the protospacer adjacent motif (PAM)-interacting (PI), HNH, and RuvC domains. Cas9 targets double-stranded DNA (dsDNA), depending on the guide RNA (gRNA) and cleaves DNA as described below^1–13^. Cas9 initially recognizes PAM in dsDNA using the PI domain. Cas9 then unwinds the target and non-target DNA strands located at the PAM-proximal region through the action of the REC lobe and RuvC domain, which is followed by annealing of the target DNA and gRNA strands (R-loop). R-loop formation triggers cleavage of dsDNA using the HNH and RuvC domains. This high programmability of gRNA has allowed the use of Cas9 for genome editing.

For the application of Cas9 in gene therapy, off–target DNA cleavage^14–23^ and low editing efficiency with sequence dependence^17,24–27^ in Cas9 have been raised as critical issues. To reduce off-target DNA cleavage, eCas9^28^ and HF1-Cas9^29^ were designed based on structure-guided protein engineering of the Cas9 protein. The reduction of off-target cleavage was clarified to be caused by enhanced proofreading or increased release of off-target DNA upon slowing DNA cleavage^30–32^.

The low editing efficiency and off-target editing may be highly coupled with the target search dynamics of Cas9. In vitro single-molecule fluorescence microscopy demonstrated that Cas9 searches for the target sequence from genomic DNA using 3D diffusion, as described below^4^. Cas9 in solution diffuses three-dimensionally, collides with and associates with DNA, and then reads the sequence by matching with the gRNA sequence. In most cases, the bound DNA is not targeted for Cas9 with gRNA, and Cas9 dissociates from the non-target DNA. Cas9 repeats these processes until Cas9 finds its target. This 3D diffusion search is supported by the single-molecule tracking of cells^33^. The search time of the target DNA by single Cas9 was estimated to be 6 h in *Escherichia coli*^34^ and 4 h in *Lactococcus lactis*^35^, which would suggest a much longer search time in human cells, with 1300-fold or 2500-fold larger size of DNA. The target search by 3D diffusion was unique to Cas9. In contrast, 1D diffusion (sliding) along DNA, as well as 3D diffusion in solution, called a facilitated diffusion mechanism, are utilized by many DNA-binding proteins in these target searches^36–38^. If Cas9 can slide along the DNA, the target search time would be reduced dramatically, thus promoting the double-strand breaks and following a subsequent reaction like non-homologous end joining (NHEJ) or homology-directed repair (HDR). This would increase the efficiency of the overall editing process (corresponding to successful editing percentage). In addition, the sliding of Cas9 along DNA could decrease off-target editing, which occurs in the paused state on DNA.

In this study, we aimed to engineer Cas9 to slide along DNA, which might facilitate genome editing and reduce off-target cleavage. We used the following two approaches: the first approach aimed to reduce the sliding friction along DNA by removing the interactions of Cas9 residues with DNA. The other aimed to facilitate the sliding motion by adding the basic tail of Nhp6A, as the sliding of Nhp6A requires the basic tail^39^.

## Results

### Non-engineered dCas9 pauses on DNA but slides a small amount

**Figure 1:**
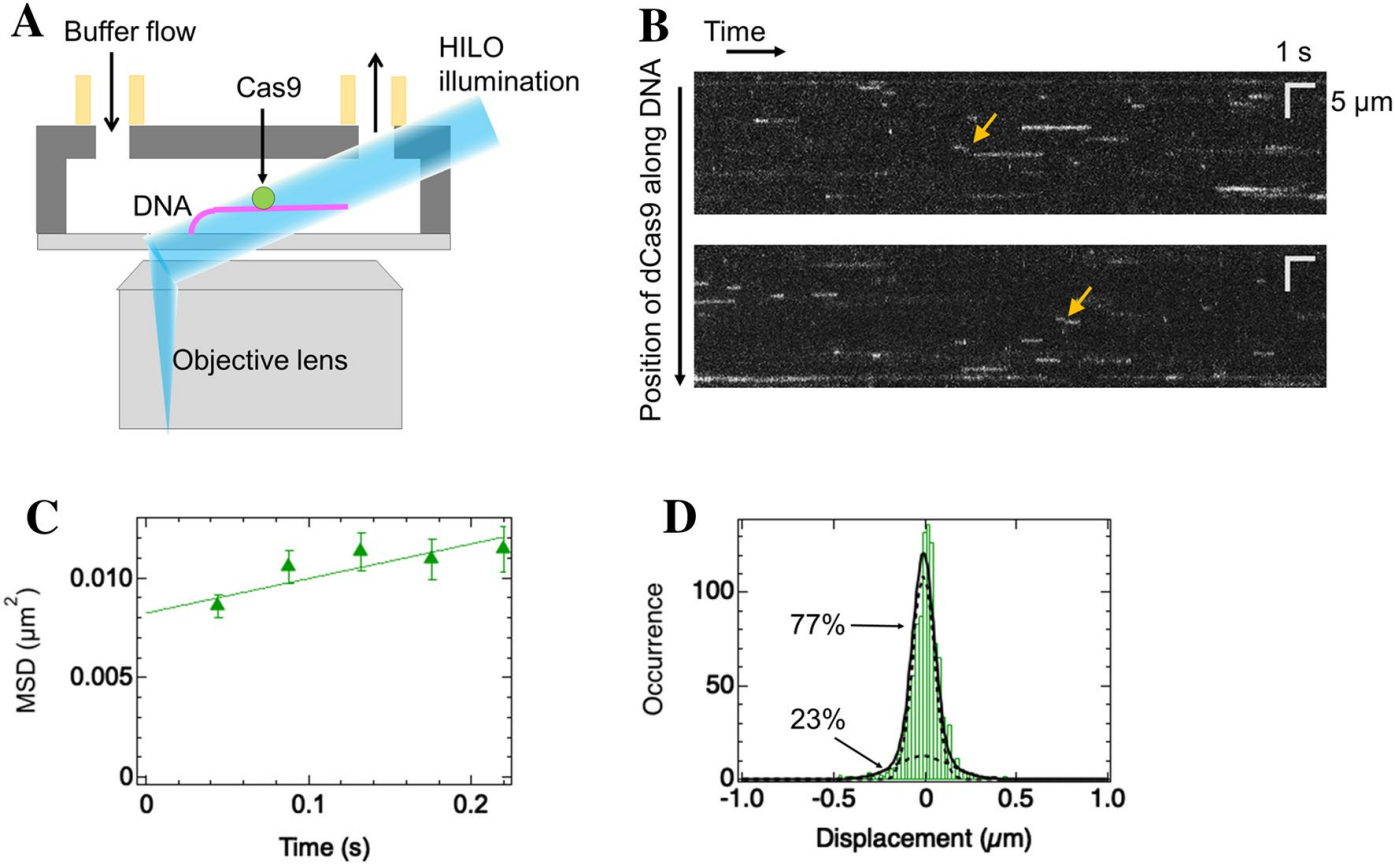
dCas9 exhibits sliding and paused modes on DNA in the absence of sgRNA. **(A)** Single-molecule tracking of dCas9 mutants labeled with ATTO488 in the flow cell. DNA (pink line) is tethered at one end and stretched by buffer flow. The labelled dCas9 mutant (green circle) is illuminated by HILO (blue sheet). The movements of dCas9 mutants bound to DNA were recorded. **(B)** Typical kymographs of dCas9. White lines or curves denote the traces of single molecules. Yellow arrows demonstrate sliding molecules. **(C)** MSD plots of all tracked molecules of dCas9 (*N* = 232). The errors denote standard errors. A best-fit linear line is shown (*R*^2^ = 0.80). **(D)** Displacement distribution of dCas9 at a time interval of 176 ms. The black curve shows the best-fit curve based on the sum of two Gaussian functions. The dashed curves represent the distributions of each mode.

Because previous studies of *Streptococcus pyogenes* Cas9 showed different results: no sliding of Cas9 labeled with Q-dot detected using fluorescence microscopy^4^ and short distance sliding of non-labeled Cas9 along DNA by atomic force microscopy^8^ in the absence of gRNA, we first measured the movement of non-engineered Cas9 along DNA using a fluorescence microscope (Fig. 1A). To simplify the system, we used deactivated Cas9 (dCas9) labeled with the fluorescent dye ATTO488 and measured the movement of the labeled dCas9 along DNA in the absence of gRNA. Because the maltose binding protein (MBP) tag suppressed the aggregation of dCas9, the subsequent measurements were mainly conducted without cleavage of the MBP tag. The DNA garden method was used to tether λ DNA in the flow cell^40^. Briefly, the DNA garden produces DNA array in which biotinylated DNAs are aligned and tethered on lines via micro-contact printing of neutravidin molecules on the flow cell surface. In this study, the DNA garden was used for high-throughput tracking of dCas9 and its engineered mutants along DNA at single molecule level. We used 50 mM potassium glutamate (KGlu) in the measurements, as some dCas9 mutants were subsequently introduced and bound to DNA to a lesser extent in 150 mM KGlu. Highly inclined and laminated optical sheet (HILO) was used to selectively illuminate molecules bound to DNA. Kymographs demonstrated that dCas9 molecules bound to DNA, and many of them were paused on DNA (Fig. 1B). The short-term movements of dCas9 along DNA were observed in a small fraction, indicating transient sliding along the DNA (arrows in Fig. 1B).

For quantitative analysis, we tracked 232 traces. The mean square displacement (MSD) plot calculated from all traces showed a slight linear increase over time, suggesting 1D diffusion of dCas9 along DNA (Fig. 1C). The average diffusion coefficient (*D*) value, calculated from the slope of the MSD corresponding to 2*D*, was 0.009 ± 0.003 μm^2^/s. The displacement distribution fitted well with the sum of the two Gaussian functions (Eq. 1), confirming the two modes of sliding and paused modes (Fig. 1D). The *D* value for the sliding mode was 0.07 ± 0.02 µm^2^/s, and the sliding percentage was 23 ± 6%. The *D* value for the paused mode was estimated to be 0.010 ± 0.001 µm^2^/s, which was within the experimental accuracy of the position of a molecule on DNA. We noted that the MBP tag did not significantly affect the sliding behavior of dCas9 (Supplementary Fig. S1). The *D* value for the sliding mode did not depend on the salt concentration, supporting 1D movement with continuous contact with DNA, rather than hopping/jumping along DNA (Supplementary Fig. S2A). Because the diffusion and percentage of the sliding mode of dCas9 were much lower than those of p53 with a similar size^41^, we were motivated to engineer dCas9 to facilitate the sliding dynamics along DNA.

### Mutational engineering of dCas9 to facilitate sliding dynamics

**Figure 2:**
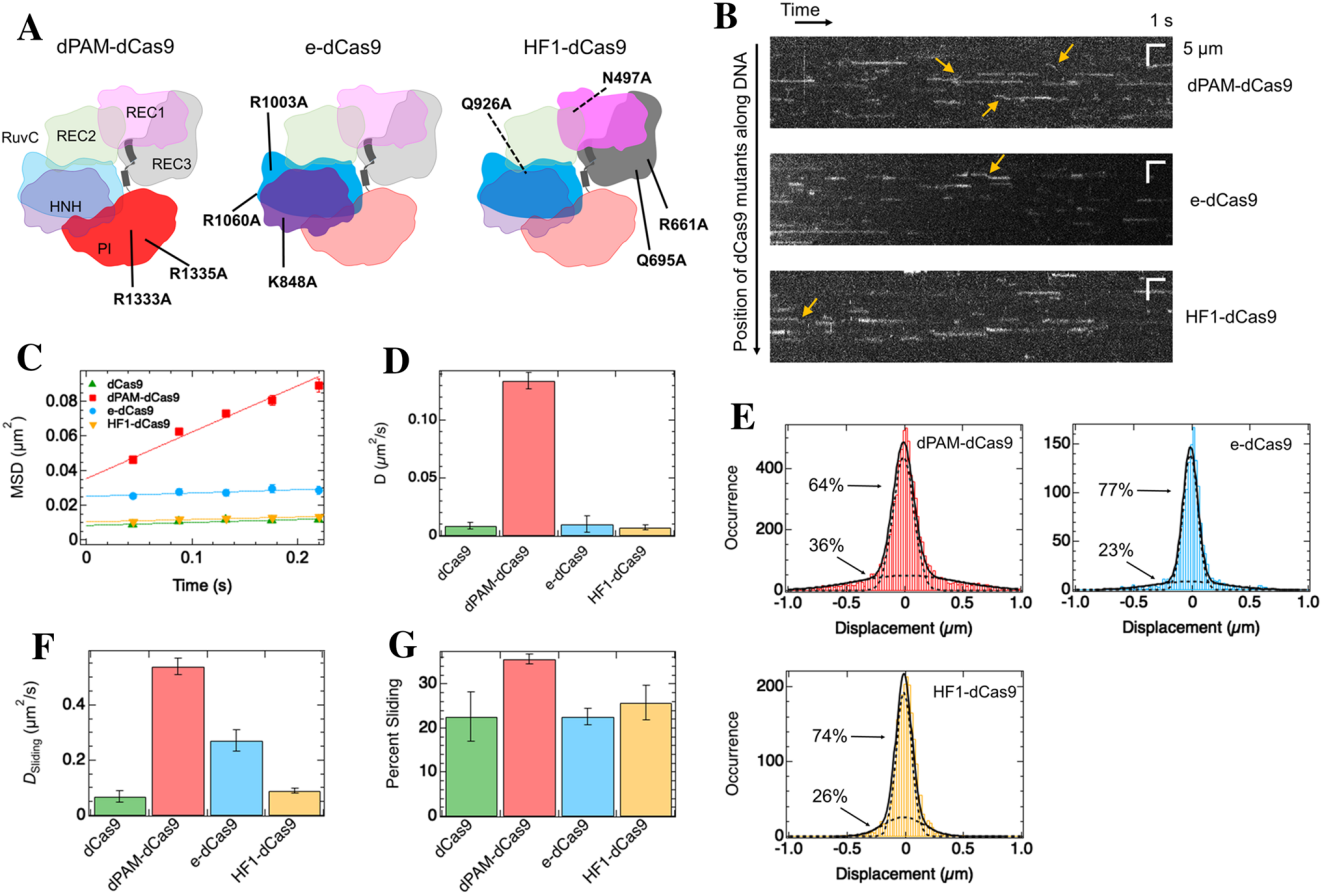
PAM-recognizing residues stabilize the sliding mode and cause high friction for the sliding in the absence of sgRNA. **(A)** Schematic diagram of mutation sites in 3D structures of dCas9 mutants. dCas9 is composed of the REC1, REC2, REC3, RuvC, HNH, and PI domains. The domains with mutation sites are displayed densely. **(B)** Typical kymographs of dCas9 mutants. White lines or curves denote traces of single molecules. Yellow arrows represent sliding molecules. **(C)** MSD plots of all tracked molecules of dCas9 and its mutants (*N* = 232 for dCas9, 1754 for dPAM-dCas9, 255 for e-dCas9, and 448 for HF1-dCas9). The errors denote standard errors. Best-fit linear lines are shown (*R*^2^ = 0.80 for dCas9, 0.88 for dPAM-dCas9, 0.72 for e-dCas9, and 0.92 for HF1-dCas9). **(D)** Average diffusion coefficient of dCas9 and its mutants along DNA. **(E)** Displacement distribution of dCas9 mutants at a time interval of 176 ms. Black curves represent the best-fit curves based on the sum of two Gaussian functions. The dashed curves represent the distributions of each mode. **(F)** Diffusion coefficient of sliding mode of dCas9 and its mutants. **(G)** Sliding mode percentage of dCas9 and its mutants. The errors denote fitting errors in panels D, F, and G.

We hypothesized that some residues of dCas9 strongly interact with DNA, generating a large amount of friction during sliding. If we replace such strongly interacting residues with others, this would facilitate the dCas9 mutant to slide along the DNA. To test this, we prepared three mutants of dCas9 that could weaken the interactions between different domains and DNA and facilitate sliding along DNA (Fig. 2A, Supplementary Fig. S3). dPAM-dCas9 lacks specific interactions of the PI domain with the PAM sequence of DNA (R1333A R1335A)^2^. An enhanced mutant of dCas9 (e-dCas9) weakens the non-specific interactions of RuvC and HNH domains with the DNA strand (K848A K1003A R1060A)^28^. A high-fidelity mutant of dCas9 (HF1-dCas9) reduces non-specific contacts of REC and RuvC domains to DNA or RNA strands (N497A R661A Q695A Q926A)^29^. The three mutants were selected as representative candidates to satisfy the following criteria: (i) multiple mutation sites, (ii) mutations located in different domains, and (iii) mutations identified to weaken the interaction to DNA.

The movement of the three mutants of dCas9 were measured using single-molecule fluorescence microscopy. Kymographs demonstrated that all three mutants possessed sliding and paused motions, and dPAM-dCas9 moved more frequently than dCas9 and the other mutants (Fig. 2B). The MSD plots confirmed the larger 1D diffusion of dPAM-dCas9 (Fig. 2C). The average *D* value of dPAM-dCas9 was 15-fold higher than that of dCas9 (Fig. 2D). In contrast, the average *D* values for e-dCas9 and HF1-dCas9 were similar to that of dCas9 (Fig. 2D). Furthermore, the displacement distributions of the three mutants fitted well to the sum of the two Gaussian functions, thus confirming the presence of the two modes (Fig. 2E). The *D* value of the sliding mode for dPAM-dCas9 was 0.54 µm^2^/s, representing a ninefold increase from that of dCas9, indicating that PAM-recognizing arginines of dCas9 interact with DNA in the sliding mode and slow the sliding along the DNA (Fig. 2F). In addition, the sliding mode percentage in dPAM-dCas9 increased 1.5-fold from that of dCas9, suggesting that specific binding to PAM sequences of λ DNA occurs in the paused mode (Fig. 2G). In contrast, e-dCas9 and HF1-dCas9 did not affect the *D* values for the two modes or the sliding percentage significantly, except for the *D* value of the sliding mode of e-dCas9. A fourfold enhancement of the *D* value for the sliding mode in e-dCas9 implies that the RuvC domain also interacts with DNA in the sliding mode (Fig. 2F). The parameters obtained for the mutants are listed in Supplementary Table S1. Overall, we found that the reduction of interactions with DNA, especially observed in the removal of specific binding to the PAM sequence, facilitated sliding by destabilizing the paused mode or reducing the friction of sliding movements.

### The basic tail of Nhp6A increases the sliding-mode population of dCas9

**Figure 3:**
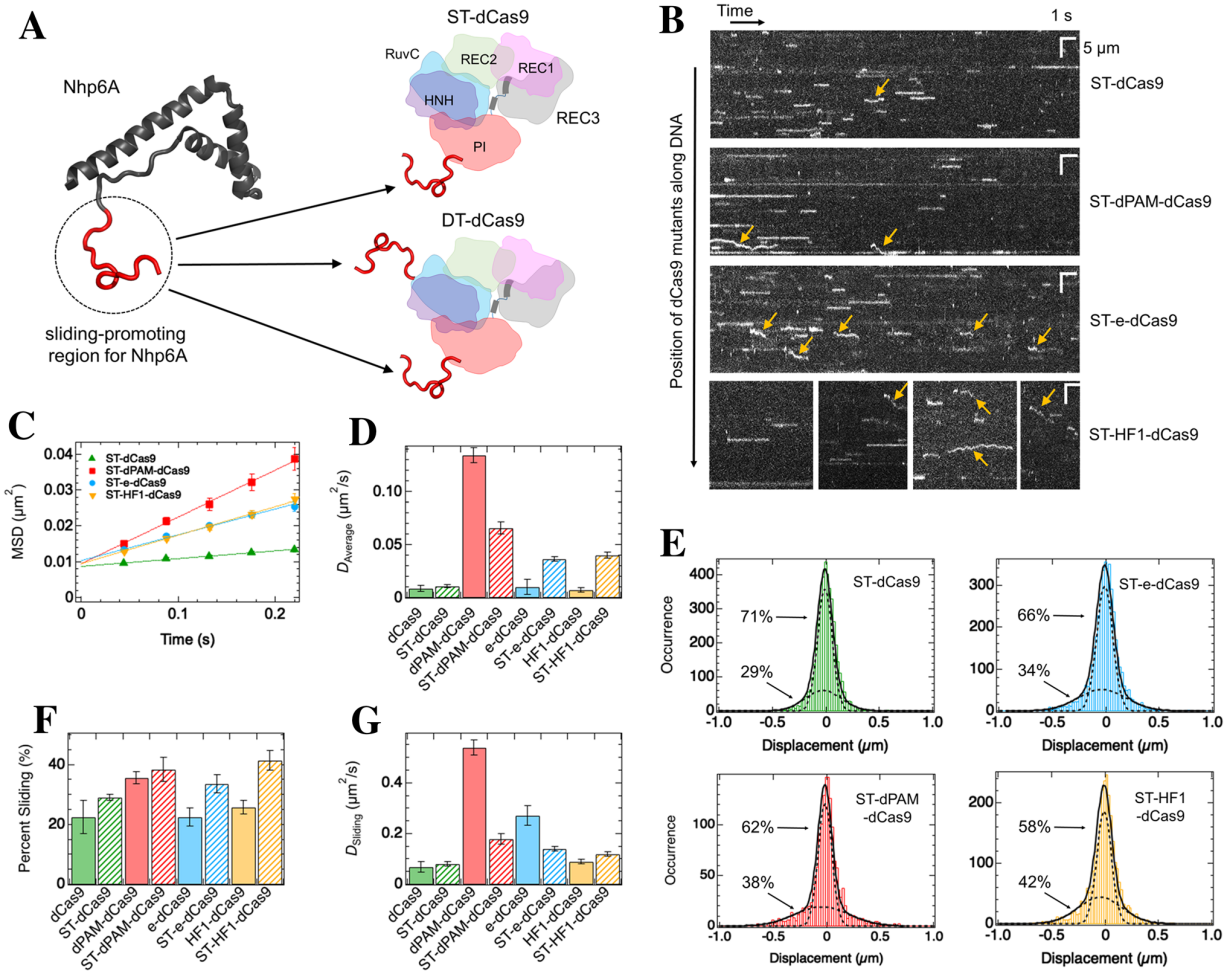
The basic tail of Nhp6A enhances the sliding mode population of dCas9 in the absence of sgRNA. **(A)** Schematic diagram of Cas9 hybrids (ST-dCas9 and DT-dCas9) with the sliding-promoting region (red) introduced from Nhp6A (PDB code 1LWM). dCas9 is composed of REC1, REC2, REC3, RuvC, HNH, and PI domains. **(B)** Typical kymographs of dCas9 hybrid mutants. White lines or curves denote the traces of single molecules. Yellow arrows show sliding molecules. **(C)** MSD plots of all tracked molecules of dCas9 hybrid mutants (*N* = 787 for ST-dCas9, 360 for ST-dPAM-dCas9, 852 for ST-e-dCas9, and 520 for ST-HF1-dCas9). The errors denote standard errors. The best-fit linear lines are shown (*R*^2^ = 0.88 for ST-dCas9, 0.81 for ST-dPAM-dCas9, 0.87 for ST-e-dCas9, and 0.82 for ST-HF1-dCas9). **(D)** Average diffusion coefficient of dCas9 hybrid mutants along DNA. **(E)** Displacement distribution of dCas9 hybrid mutants at a time interval of 176 ms. Black curves are the best-fit curves based on the sum of two Gaussian functions. The dashed curves represent the distributions of each mode. **(F)** Sliding mode percentage of dCas9 hybrid mutants. **(G)** Diffusion coefficient of the sliding mode for dCas9 hybrid mutants. In panels D, F, and G, the errors denote fitting errors, and the data of dCas9 and its mutants are displayed for comparison.

To further speed up the sliding of dCas9, we next focused on the basic disordered region (residues 2–16) of Nhp6A that could significantly facilitate sliding along DNA. This is based on the fact that the removal of the disordered region inhibited the sliding by Nhp6A^39^. We hypothesized that the attachment of the disordered region from Nhp6A to dCas9 could facilitate sliding in a similar way. To test this, we prepared two dCas9 hybrids connected to the disordered region of Nhp6A: dCas9 with a single tail at the C-terminus (ST-dCas9) and dCas9 with double tails at the N- and C-termini (DT-dCas9) (Fig. 3A). DT-dCas9 molecules easily formed irreversible collapsed aggregates of DNA in the absence of flow, and the DNA was not stretched back in the presence of flow, which prevented the single-molecule tracking of DT-dCas9 (Supplementary Fig. S4). In contrast, ST-dCas9 exhibited this irreversible aggregation formation of DNA at a much lower frequency, similar to the other mutants of dCas9. Considering these facts, we chose to focus on the ST-dCas9 mutant. The kymograph of ST-dCas9, similar to dCas9, demonstrated sliding and paused motions in 50 mM KGlu (Fig. 3B). The average *D* value of ST-dCas9, estimated from the MSD plot, was 1.3-fold higher than that of dCas9 (Fig. 3C,D, Supplementary Table S1). The displacement distribution of ST-dCas9 was well fitted to the sum of double Gaussians, confirming the presence of sliding and paused modes (Fig. 3E). The 1.3-fold enhanced diffusion, on average, might be attributed to the small enhancement of the *D* value and percentage of the sliding mode (Fig. 3F,G, Supplementary Table S1). We confirmed the sliding of ST-dCas9 based on the absence of a systematic increase in the *D* value of the sliding mode with the salt concentration (Supplementary Fig. S2B).

We next investigated the effect of ST on the 1D diffusion of dCas9 mutants with a weakened affinity for DNA. To this end, we prepared three dCas9 hybrid mutants connected to the disordered region of Nhp6A at the C-termini: e-dCas9 with a single tail (ST-e-dCas9), HF1-dCas9 with a single tail (ST-HF1-dCas9), and dPAM-dCas9 with a single tail (ST-dPAM-dCas9) (Supplementary Fig. S3). The kymographs of the three mutants demonstrated sliding and paused motion in 50 mM KGlu (Fig. 3B). The average *D* value of ST-e-dCas9, obtained by MSD analysis, was 3.7-fold higher than that of e-dCas9 (Fig. 3C,D). In addition, the average *D* value of ST-HF1-dCas9 was 5.4-fold higher than that of HF1-dCas9 (Fig. 3C,D). For ST-e-dCas9 and ST-HF1-dCas9, the displacement distributions were well fitted to the sum of the double Gaussians, confirming the presence of sliding and paused modes (Fig. 3E). The attachment of ST enhanced the percentage of the sliding mode to 1.5-fold for e-dCas9 and 1.6-fold for HF1-dCas9 (Fig. 3F). The *D* value of the sliding mode for ST-HF1-dCas9 increased 1.4-fold from that of HF1-dCas9, whereas that for ST-e-dCas9 decreased 1.9-fold from that of e-dCas9 (Fig. 3G).

Contrary to ST-e-dCas9 and ST-HF1-dCas9, the average *D* value of ST-dPAM-dCas9 decreased twofold from that of dPAM-dCas9 (Fig. 3C,D). The displacement distribution analysis of ST-dPAM-dCas9 demonstrated a threefold reduction in the *D* value in the sliding mode, with a comparable sliding percentage, upon attachment of ST (Fig. 3E–G). Taken together, the attachment of ST to dCas9 mutants modulated the sliding dynamics of dCas9.

### sgRNA modulates the effect of the basic tail and mutations on the sliding of dCas9

**Figure 4:**
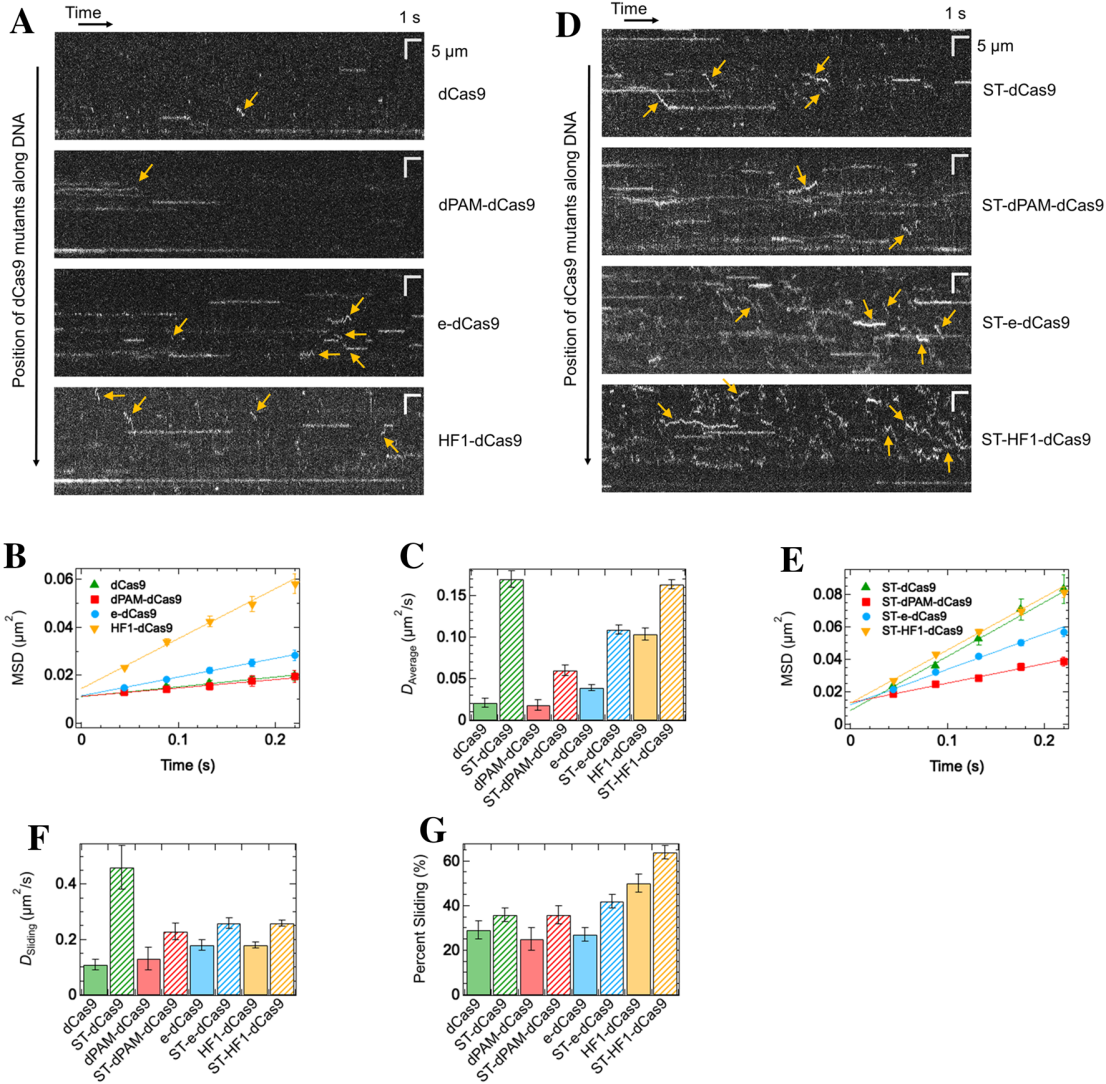
The basic tail of Nhp6A and mutations enhance the sliding of dCas9 in the presence of sgRNA. **(A)** Typical kymographs of dCas9 mutants. **(B)** MSD plots of all tracked molecules of dCas9 mutants (*N* = 224 for dCas9, 153 for dPAM-dCas9, 403 for e-dCas9, and 519 for HF1-dCas9). Best-fit linear lines are shown (*R*^2^ = 0.87 for dCas9, 0.81 for dPAM-dCas9, 0.82 for e-dCas9, and 0.82 for HF1-dCas9). **(C)** Average diffusion coefficient of dCas9 mutants, including hybrids, along DNA. **(D)** Typical kymographs of dCas9 hybrid mutants. Typical sliding molecules are indicated by yellow arrows for ST-e-dCas9 and ST-HF1-dCas9 because of too many sliding events. **(E)** MSD plots of all tracked molecules of dCas9 hybrid mutants (*N* = 209 for ST-dCas9, 342 for ST-dPAM-dCas9, 633 for ST-e-dCas9, and 1008 for ST-HF1-dCas9). The best-fit linear lines are shown (*R*^2^ = 0.74 for ST-dCas9, 0.85 for ST-dPAM-dCas9, 0.85 for ST-e-dCas9, and 0.83 for ST-HF1-dCas9). **(F)** Diffusion coefficient of the sliding mode for dCas9 mutants, including hybrids. **(G)** Sliding mode percentage of dCas9 mutants, including hybrids. In panels A and D, white lines or curves denote the traces of single molecules, and yellow arrows show sliding molecules (selected for ST-e-dCas9 and ST-HF1-dCas9 because of too many sliding events). In panels B and E, the errors denote standard errors.

Because Cas9 functions to form a complex with gRNA, we examined the effect of mutations and basic tails on the sliding dynamics of dCas9 and sgRNA complexes. sgRNA was designed to target the 12,128^th^-12,147th bases of λDNA, following the methods of two prior reports^4,32^ with some modifications. The gel shift assay confirmed that the purified sgRNA formed a complex with dCas9 or ST-dCas9 and 55-bp target DNA labeled with 6-FAM, which was detected as a band shift from the complex without sgRNA (Supplementary Fig. S5). This is consistent with previous reports^1,4^. For single-molecule measurements, the complex of dCas9 and sgRNA was formed after 10 min of incubation and then introduced into the flow cell with a DNA array. dCas9 molecules at 1 nM, twofold higher concentrations used in the absence of sgRNA, were detected on DNA, suggesting that sgRNA reduces affinity by altering the tertiary complex structure (Fig. 4A). dCas9 molecules were associated with random positions of λDNA, including the target located one-fourth from the tethered end, suggesting that the target binding event was masked by many non-specific binding events (Fig. 4A). This is consistent with a report by Sternberg et al.^4^. Accordingly, the observed dynamics were assumed to be mainly attributed to non-specific associations, rather than specific associations. The MSD plot showed a linear relationship with time, confirming the diffusion of the dCas9 and sgRNA complex (Fig. 4B). The average *D* value was 2.3-fold higher than that in the absence of sgRNA. The displacement distribution analysis demonstrated the sliding and paused modes of the dCas9 and sgRNA complexes (Supplementary Fig. S6, Supplementary Table S2).

We next investigated the effect of dCas9 mutations on sliding dynamics in the presence of sgRNA. For dPAM-dCas9 introduced at 2 nM, the number of molecules bound to DNA decreased from that of dCas9, implying that PAM-interacting residues enhanced the binding affinity (Fig. 4A). Most dPAM-dCas9 molecules were stationary and slid only a small fraction. The average *D* value of dPAM-dCas9, obtained from the MSD plots, was comparable to that of dCas9 (Fig. 4B,C). The sliding of e-dCas9, introduced at 1 nM, was observed more frequently than that of dCas9, but stationary molecules were also observed. The average *D* value of e-dCas9 was 1.9-fold higher than that of dCas9 (Fig. 4B,C). HF1-dCas9 showed sliding with a short residence time in a higher fraction, as well as pausing with a long residence time (Fig. 4A). The average *D* value of HF1-dCas9 was 5.0-fold higher than that of dCas9 (Fig. 4B,C). The e- and HF1- mutations enhanced the sliding dynamics of the dCas9 and sgRNA complex, whereas these mutations did not affect dCas9 in the absence of sgRNA (Figs. 2D and 4C). In contrast, dPAM mutations did not affect the sliding dynamics of the dCas9 and sgRNA complex, whereas it enhanced that of dCas9 in the absence of sgRNA (Figs. 2D and 4C). The opposite effects of mutations on sliding in the presence and absence of sgRNA demonstrates that the conformational change of dCas9, which is induced by sgRNA, dramatically alters the interaction between dCas9 and DNA.

We further examined the effect of ST introduced into dCas9 mutants on sliding dynamics in the presence of sgRNA. ST-dCas9 molecules showed short-lived sliding, in addition to a relatively long pausing (Fig. 4D). The average *D* value of ST-dCas9 increased 8.1-fold from that of dCas9 (Fig. 4C,E). For ST-dPAM-dCas9 introduced at 1 nM, the number of molecules bound to DNA increased as compared to that of dPAM-dCas9, indicating that ST enhanced the binding affinity (Fig. 4D). Additionally, ST-dPAM-dCas9 exhibited both sliding and pausing. The average *D* value of ST-dPAM-dCas9 was 3.3-fold higher than that of dPAM-dCas9 (Fig. 4C,E). ST-e-dCas9 and ST-HF1-dCas9 showed sliding with a short residence time in a higher fraction, as well as pausing with a long residence time (Fig. 4D). The average *D* values of ST-e-dCas9 and ST-HF1-dCas9 were enhanced 2.8-fold and 1.6-fold from that of e-dCas9 and HF1-dCas9, respectively (Fig. 4C,E). Altogether, the attachment of ST to dCas9 promoted the sliding of the dCas9 and sgRNA complexes along the DNA.

To clarify the mechanism behind the enhanced sliding in the presence of sgRNA, the displacement distributions were analyzed. In all mutants of dCas9 including hybrids, the displacement distributions were well described by the sum of the sliding and paused modes (Supplementary Fig. S6). The enhancement of the average *D* values was attributed to the increase in the *D* value or the percentage of the sliding mode (Fig. 4F,G, and Supplementary Table S2). In particular, ST-dCas9 significantly enhanced the diffusion of the sliding mode, rather than the population of the sliding mode, as compared to dCas9. ST-HF1-dCas9 promoted both diffusion and population of the sliding mode from that of dCas9. Thus, the removal of interacting residues or the attachment of ST might alter the complex conformation of the sliding mode, thus promoting the diffusion of engineered Cas9 along DNA.

## Discussion

In this study, we tested two approaches to engineer Cas9 to slide along DNA, which may facilitate genome editing and reduce off-target cleavage. The first approach reduced the sliding friction along DNA by removing the interactions of Cas9 residues with DNA. This is supported by the fact that some key residues were identified to retard the sliding in glycosylases^42,43^. When strongly interacting residues are mutated, sliding is facilitated by the weakening of the sliding friction. The second approach is to facilitate sliding by introducing a sliding-promoting peptide found in other DNA-binding proteins. In fact, the deletion of basic tails reduced the sliding of p53^44,45^ and Nhp6A^39^; these basic tails are considered to be sliding-promoting peptides. Here, we used the sliding-promoting peptide identified in Nhp6A, as Nhp6A functions as a monomer like Cas9, whereas p53 acts as a tetramer. This concept is similar to that of molecular sleds^46–48^. Using the proposed approaches, we succeeded in improving the sliding ability of Cas9 (1,779 residues including MBP) to that of the p53 tetramer, a tumor-suppressing protein with a similar size (1572 residues)^40,41^.

A series of single-molecule data for measuring the action by engineering provided a molecular model for the sliding of Cas9 along DNA. In particular, the tertiary structure of the Cas9/DNA or Cas9/gRNA/DNA complex with non-complementary sequences has not been determined, likely owing to the non-uniform conformation or kinetically transient intermediates. In this study, key interactions were identified in the paused and sliding modes of Cas9. For apo-Cas9 without gRNA, the lack of PAM interactions significantly promoted the diffusion and population of the sliding mode (Fig. 2F,G). This implies that apo-Cas9 in the sliding mode moves along DNA with contact mediated by the PI domain (R1333 R1335), which functions as a sensor of the PAM sequence. In addition, this interaction with PAM becomes tighter in the paused mode than in the sliding mode, further stabilizing the paused mode. For Cas9 with gRNA, the conformational change of Cas9 induced by gRNA^49,50^ altered the key interactions. The e- and HF1-mutation sites, located in the RuvC, HNH, and REC domains (N497 R661 Q695 K848 Q926 K1003 R1060), are involved in the sliding mode, based on the enhanced diffusion upon the removal of these interactions (Fig. 4F). Furthermore, HF1 mutation sites (N497 R661 Q695 Q926) participate in the stabilization of the paused mode (Fig. 4G). In e-Cas9 and HF1-Cas9, the reduced off-target effect is considered to enhance proofreading and release of off-target DNA^30–32^. Our data suggest another reason for the reduced off-target effect in which the HF1-mutations reduce the population in the paused mode, which is considered to be prone to off-target binding.

**Figure 5:**
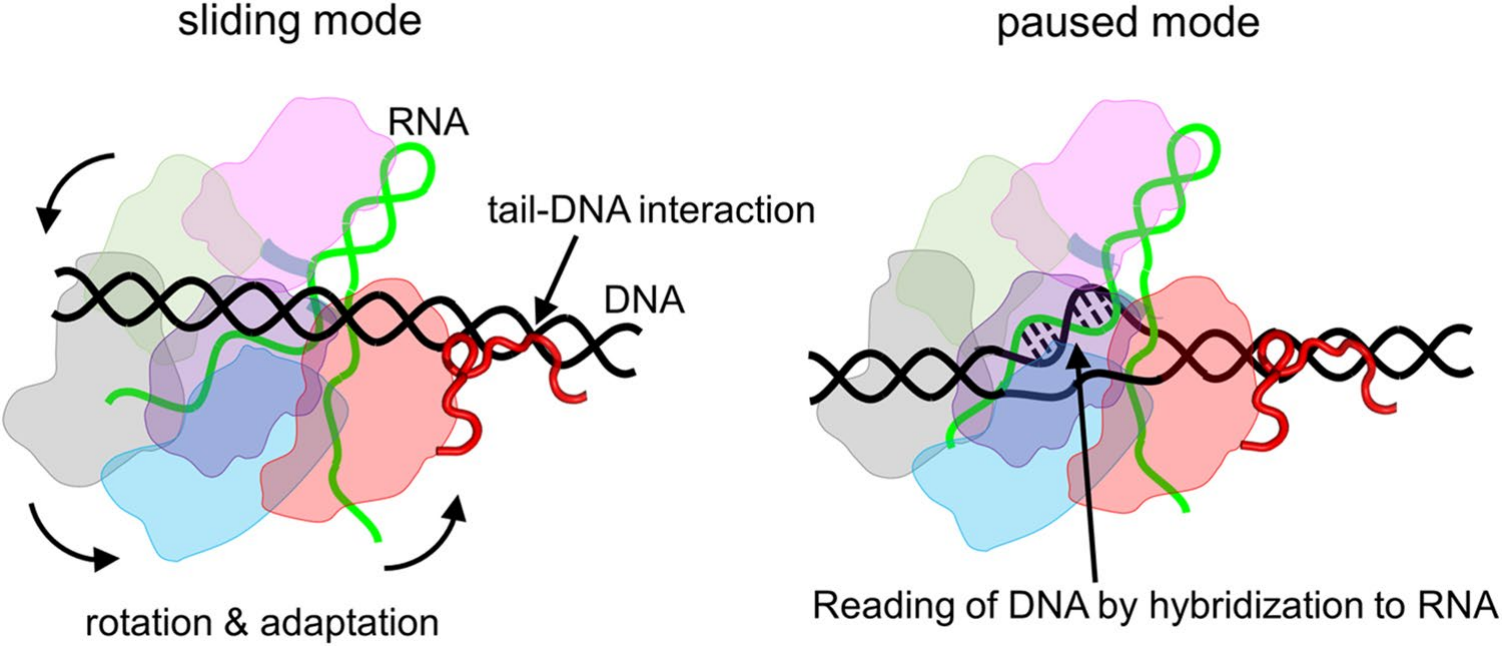
Schematic model of sliding and paused modes of the Cas9 hybrid with tail. Cas9 is composed of REC1 (light pink), REC2 (light green), REC3 (light grey), RuvC (light blue), HNH (light purple), and PI (light red) domains. Green and black strands denote gRNA and DNA, respectively. In sliding mode, the tail (red), introduced into Cas9, interacts with DNA, triggering the rotation of the Cas9 structure. This conformational change causes the adaptation of the complex structure, facilitating the sliding dynamics along DNA. In paused mode, Cas9 induces unwinding of DNA, and subsequent partial hybridization between DNA and gRNA strands occurs. This action enables Cas9 to read the sequence of DNA.

Introducing a sliding-promoting tail from Nhp6A dramatically affects the interactions between Cas9 and DNA in the sliding mode as compared to those of the paused mode. The introduced tail interacts with DNA with a relatively high affinity in a non-sequence-specific manner, which likely pulls the PI domain of Cas9 and rotates the overall Cas9 structure with gRNA on DNA (Fig. 5). This action could trigger the adaptation of the complex structures: the interactions between the PI domain and PAM sequence are weakened and the interactions relevant to e- and HF1-mutation sites are strengthened, thereby lowering the sliding friction (Fig. 5). In paused mode, Cas9 seems to induce unwinding of DNA, followed by partial hybridization between DNA and gRNA strands (Fig. 5). This mode enables Cas9 to read the DNA sequence. The relatively tight complex structure in the paused mode might be less affected by the interaction between the tail and DNA than that in the sliding mode. Accordingly, we propose that the introduced tail effectively alters the Cas9 complex structure in the sliding mode, which facilitates sliding.

The Cas9 engineered in this study possesses enhanced sliding ability, which could alter the speed and efficiency of the target search, thus affecting genome editing. Non-engineered Cas9 mainly uses 3D diffusion for target search and binding, which is time-consuming^33,34^. In contrast, the engineered Cas9 would reach the target site in the genomic DNA in a shorter time, according to a facilitated diffusion mechanism involving a combination of 3D diffusion and 1D sliding^36–38,51^. However, the enhanced sliding may cause failure in the recognition of the target sequence coded on DNA: i.e., the faster the Cas9 slides along DNA, the lower the frequency at which it recognizes the target. In the kymographs, we noticed that many molecules of ST-HF1-dCas9 with sgRNA bypassed the target sequence located on λDNA. Such bypass events over the target were also observed in p53, and the target recognition probability was coupled with activity^52^. In addition, engineering of zinc finger protein Egr-1 demonstrated a tradeoff between affinity and speed^53^. Considering these facts, achieving a balance between target recognition efficiency and sliding speed is a key factor in improving the global function efficiency of Cas9 and will be a challenge for the future. If the engineered Cas9 with high sliding and recognition ability introduces double-strand breaks of the target site faster than non-engineered Cas9 in cells, the subsequent NHEJ or HDR would be started earlier, reducing the editing time and increasing the successful editing percentage.

## Methods

### Preparation of the Cas9 mutants

For dCas9, we used 10 × His-MBP-TEV-dCas9 (M1C, D10A, C80S, H840A C574S; Addgene 60815). TEV denotes the cleavage sequence of the TEV protease. Based on the dCas9, we prepared three mutants: dPAM-dCas9 (R1333A R1335A), e-dCas9 (K848A K1003A R1060A)^28^, and HF1-dCas9 (N497A R661A Q695A Q926A)^29^. The genes dPAM-dCas9, e-dCas9, and HF1-dCas9 were generated by fusing the mutated DNA fragments with the dCas9 vectors cleaved by BamHI, KpnI, and NheI using the In-Fusion HD Cloning Kit (Clontech, Mountain View). ST-dCas9 was prepared by adding 2–16 residues of Nhp6A (VTPREPKKRTTRKKK) to the C-terminus of dCas9. The ST-dCas9 gene was generated using the KOD-Plus mutagenesis kit (TOYOBO). DT-dCas9 was further prepared by adding 2–20 residues of Nhp6A (VTPREPKKRTTRKKKDPNA) to ST-dCas9 at the N-terminus. The DT-dCas9 gene was generated by Nsi I cleavage of the vector, annealing of oligonucleotides phosphorylated by T4 polynucleotide kinase (TOYOBO), and ligation by Ligation high (TOYOBO). ST-dPAM-dCas9, ST-e-dCas9, and ST-HF1-dCas9 were generated from ST-dCas9 using the methods described above.

dCas9 mutants with the MBP tag were expressed in Rosseta2(DE3) plysS cells. The cells were cultured in 4 L of 2 × YT medium at 37 °C. After the OD_600_ of the culture reached 0.6, IPTG was added to a final concentration of 0.2 mM, and the cells were cultured at 18 °C for 16 h and harvested by centrifugation. The pellet was resuspended in a buffer containing 20 mM Tris, 1 M NaCl, 1 mM TCEP, and a protease inhibitor cocktail (cOmplete, Mini; Sigma-Aldrich) (pH 8.0) and lysed by sonication on ice. After adding 10 mM MgCl_2_ and 10 U/mL DNase, the lysed cells were further incubated at room temperature for 1 h. The supernatant was collected after centrifugation. dCas9 mutants with an MBP-tag were purified using a HisTrap column (HisTrap FF crude; GE Healthcare) in a solution containing 20 mM Tris, 250 mM NaCl, 10% glycerol, and 100 or 250 mM imidazole (pH 8.0). For the cleavage of the MBP-tag, dCas9 with MBP-tag was incubated with TEV protease (Accelagen) during dialysis, in a solution containing 20 mM HEPES, 150 mM KCl, 10% glycerol, and 1 mM TCEP (pH 7.5) at 4 °C overnight. dCas9 mutants with or without the MBP-tag were further purified using an ion exchange column (HiTrap SP; GE Healthcare) in a solution containing 20 mM HEPES, 0.1–1 M KCl, 10% glycerol, and 1 mM TCEP (pH 7.5). Purity was confirmed using sodium dodecyl sulfate–polyacrylamide gel electrophoresis. The samples were stored at -80ºC. The purified mutants were labeled with ATTO488 using maleimide chemistry. The mutants labeled with ATTO488 were purified using an an ion exchange column (HiTrap SP). The labeling ratios were determined to be 0.55–1.1 dye for dCas9 mutants.

### Preparation of sgRNA and gel shift assays

We prepared sgRNA following the protocol described in a previous study^32^ with some modifications. Oligonucleotides used as templates for in vitro transcription were purchased from Eurofins (Supplementary Table S3). The template was prepared by mixing equimolar concentrations of complementary oligonucleotides in 10 mM Tris–HCl, 50 mM NaCl, and 1 mM EDTA (pH 8.0), heating to 95 °C for 5 min, and then cooling slowly. The sgRNA was synthesized using the HiScribe T7 Quick High Yield RNA Synthesis Kit (New England Biolabs), further purified using a Monarch RNA Cleanup Kit (New England Biolabs), and stored at -80 °C. Before the assays, the purified sgRNA was refolded by heating to 95 °C and then cooled slowly in 10 mM Tris–HCl, 50 mM NaCl, and 1 mM EDTA (pH 8.0).

For gel shift assays, 100 nM dCas9 or ST-dCas9 and 100 nM sgRNA were incubated for 10 min at 21 °C in a solution containing 20 mM Tris, 100 mM KCl, 10% glycerol, 1 mM DTT, and 0.5 U/μL RNasin (pH 8.0). Then, 50 nM dsDNA labeled with 6-FAM (5’-AGCAGAAATCTCTGCTGACGCATAAAGATGAGACGCTGGAGTACAAACGTCAGCT-3’; Sigma-Aldrich) was added and incubated for 10 min at 37 °C. Electrophoresis was conducted at 200 V in an 8% native acrylamide gel at 4 °C.

### Single-molecule fluorescence measurements

To tether λ DNA in the flow cell, we used DNA garden methods to produce arrays of stretchable DNA^40^. λDNA contains many specific PAM sequences for Cas9. The flow cell was placed above the objective lens in an inverted fluorescence microscope (IX-73; Olympus) with a total internal reflection fluorescence unit (IX3RFAEVAW; Olympus)^39,54^. The labeled dCas9 mutants in the flow cell were illuminated using HILO with a 488-nm laser, and the fluorescence was measured using an EM-CCD (iXon Ultra 888; Andor). We introduced dCas9 mutants with or without sgRNA (0.32 nM) in a buffer containing 20 mM HEPES, 1 mM EDTA, 0.5 mM DTT, 0.5 mg/mL bovine serum albumin, 2 mM Trolox, and 50 mM KGlu (pH 7.9) into the flow cell using a syringe pump. To form the complex with sgRNA, a solution of 100 nM dCas9 mutants and 1 μM sgRNA was incubated for 10 min at 21 °C in a solution containing 100 mM Tris, 500 mM NaCl, and 10 mM EDTA (pH 8.0), before introducing into the flow cell. Images (300 × 800 pixels) were taken at 44 ms intervals at a flow rate of 0.5 mL/min at 21 °C.

The fluorescent spots of single dCas9 mutant molecules were tracked from sequential images using ImageJ software with the plugin “Particle track and analysis”. To remove nonspecific adsorption of the fluorescent molecules on the flow cell surface, we selected trajectories using our in-house program^39^ with some modifications. To remove molecules adsorbed on the surface from the analysis, we selected traces with a local MSD larger than 5,070 nm^2^ for the perpendicular axis against the stretched DNA. In addition, we selected trajectories with at least six consecutive points. MSD plots were fitted using a linear function, 2*Dt* + *a*, where *D*, *t*, and *a* are the average diffusion coefficient, time, and offset corresponding to spatial resolution, respectively. Displacements, δ*x*, were calculated from all pairs of positions of a molecule at time intervals of 176 ms for all trajectories (δ*x* = *x*(*t* + δ*t*) − *x*(*t*), where *x* and δ*t* represent the position of molecules along DNA and time interval, respectively). To fit the displacement distribution analysis, we used the following equation:

1 $$P\left( {\delta x} \right) = \mathop \sum \limits_{{i = 1}}^{2} \frac{{A_{i} }}{{\sqrt {4\pi D_{i} \delta t} }}\exp \left( { - \frac{{\left( {\delta x + v_{i} \delta t} \right)^{2} }}{{4D_{i} \delta t}}} \right)~,$$

where *P*(δ*x*), *A*_*i*_, *v*_*i*_, and *D*_*i*_ represent the occurrence of δ*x*, amplitude of the *i*th mode, drift velocity of the *i*th mode, and diffusion coefficient of the *i*th mode, respectively. Note that δ*x* = *x*(*t*) −* x*(*t*).

## Supplementary Information

Supplementary Information.
