# Supplementary material for: Engineering of the genome editing protein Cas9 to slide along DNA
Source: Sci Rep. 2021 Jul 8;11:14165. doi: 10.1038/s41598-021-93685-9 (PMC8266852; doi:10.1038/s41598-021-93685-9)
Supplement: Supplementary file 1 — Supplementary Information. [file 41598_2021_93685_MOESM1_ESM.docx]

Supplementary Information for

**Engineering of the genome editing protein Cas9 to slide along DNA**

Trishit Banerjee^1,2^, Hiroto Takahashi^1^, Dwiky Rendra Graha Subekti^1,2^, and Kiyoto Kamagata^1,2,*^

^1^Institute of Multidisciplinary Research for Advanced Materials, Tohoku University, Katahira 2-1-1, Aoba-ku, Sendai 980-8577, Japan

^2^Department of Chemistry, Graduate School of Science, Tohoku University, Sendai 980-8578, Japan

^*^Corresponding author: Kiyoto Kamagata.

Institute of Multidisciplinary Research for Advanced Materials, Tohoku University, Katahira 2-1-1, Aoba-ku, Sendai 980-8577, Japan

Tel.: +81-22-217-5843

Fax: +81-22-217-5842

E-mail: kiyoto.kamagata.e8@tohoku.ac.jp


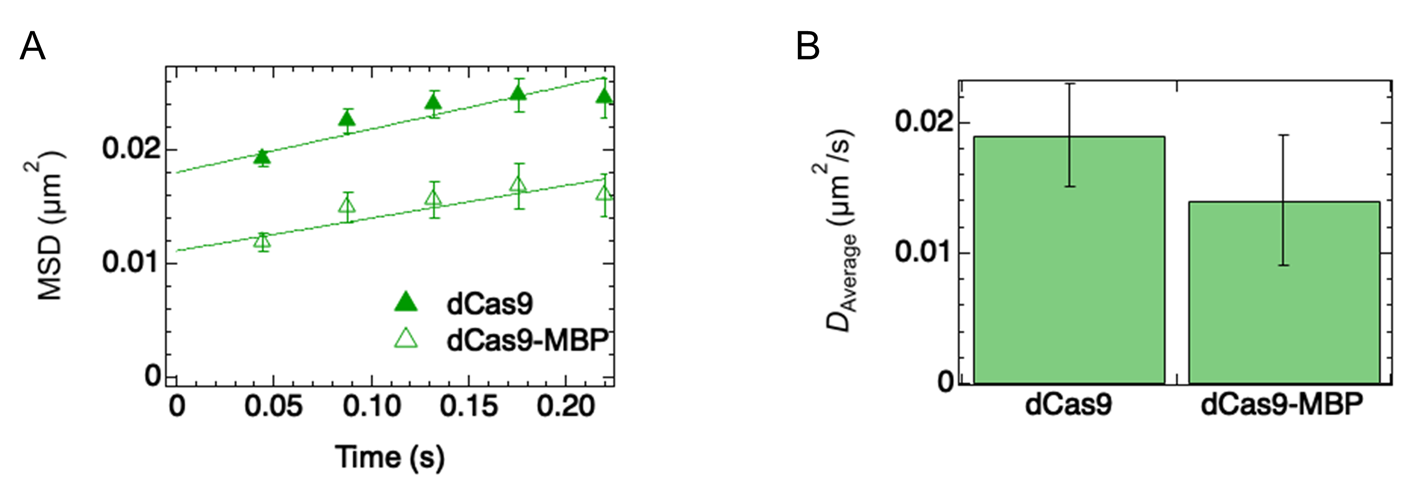


**Fig. S1.** **(A)** MSD plots of tracked molecules of dCas9 with and without an MBP-tag. The error bars denote standard errors. Best-fit linear lines are shown. Experiments were performed in 150 mM KGlu. **(B)** Average diffusion coefficient of dCas9 with and without MBP-tag along DNA.


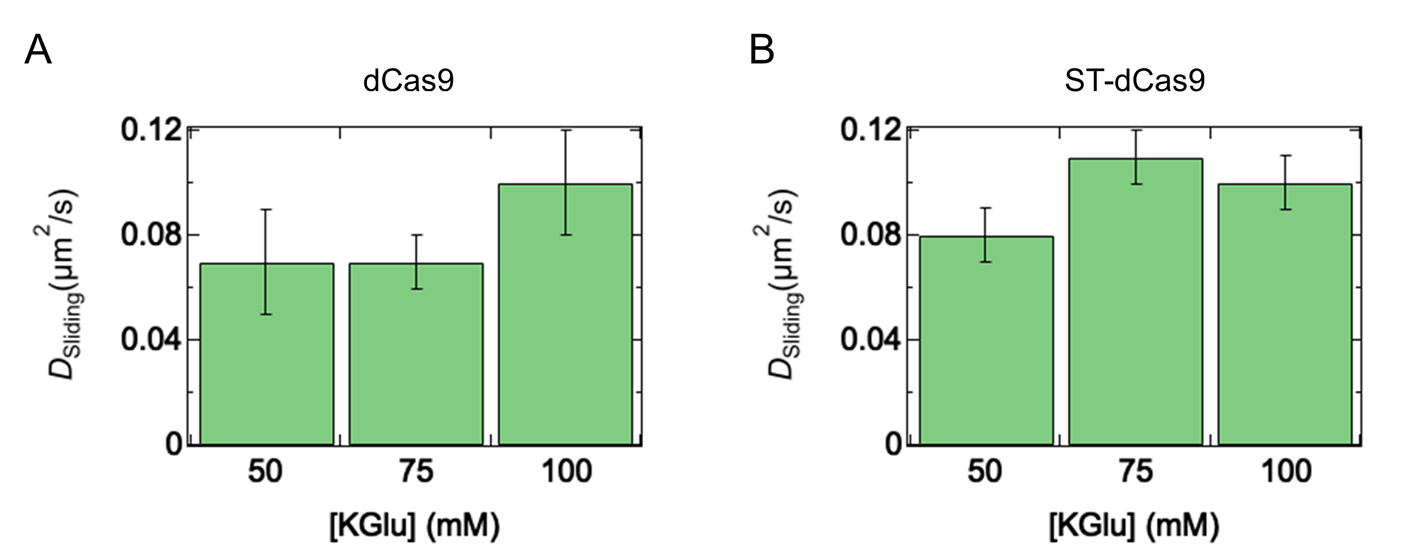


**Fig. S2.** Salt-dependence of diffusion coefficient of the sliding mode for dCas9 **(A)** and ST-dCas9 **(B)**. The diffusion coefficients were determined by fitting the displacement distribution of dCas9 mutants by the sum of two Gaussian functions. The error bars denote the fitting errors.


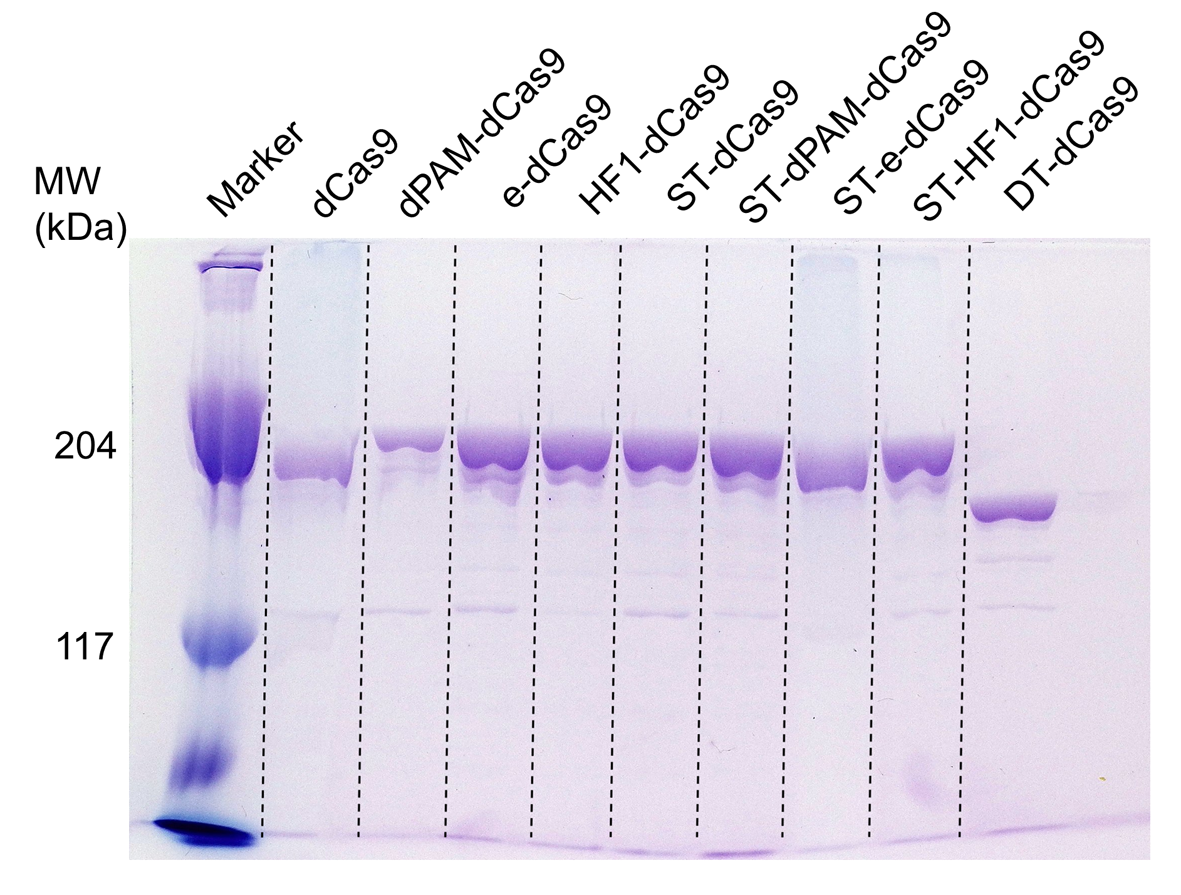


**Fig. S3.** SDS-PAGE of the dCas9 mutants used in this study


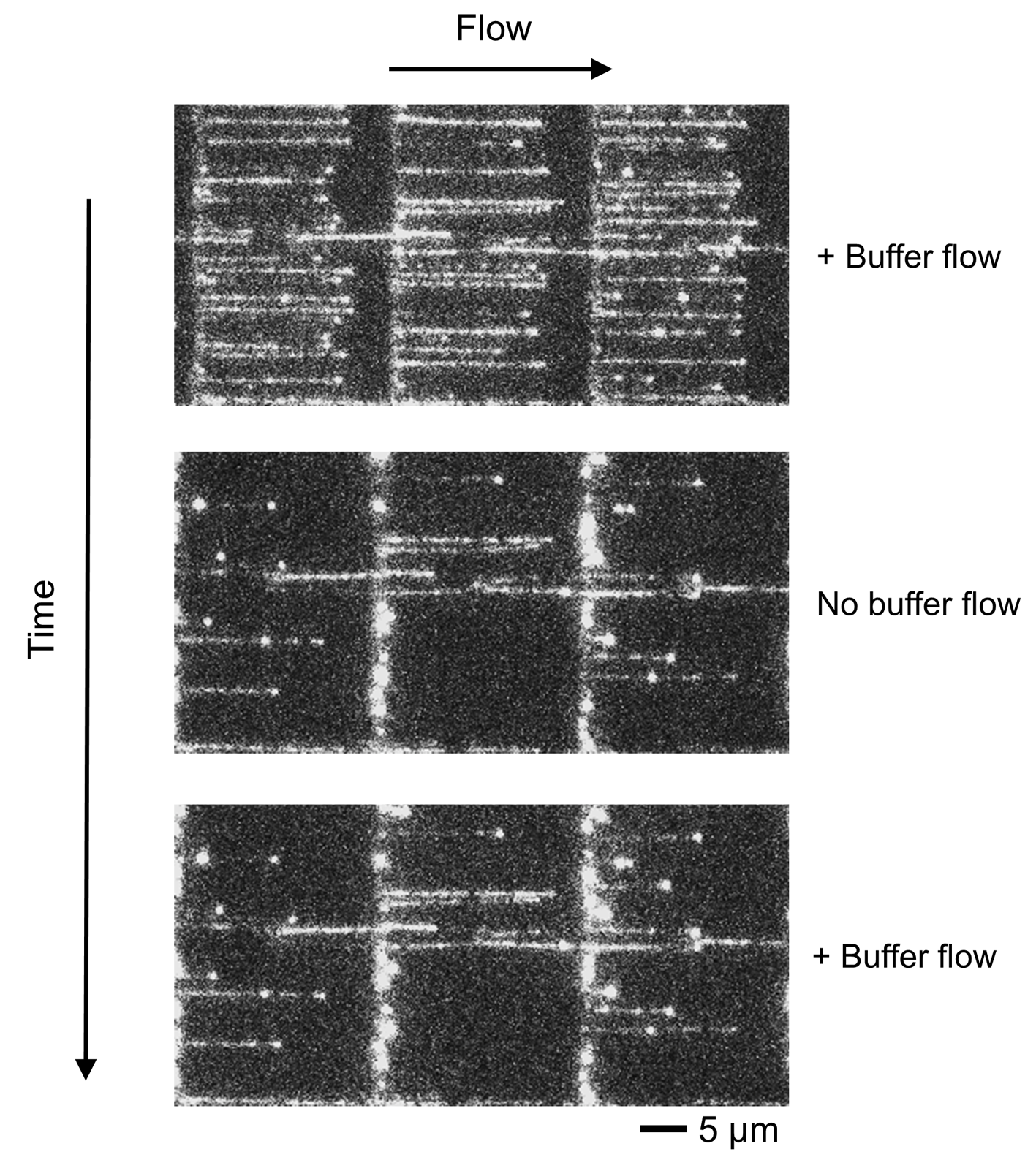


**Fig. S4.** DT-dCas9 induced collapsed aggregates of DNA in the cycles between stretching and relaxing of DNA. DT-dCas9 at 2 nM was introduced in a buffer containing 20 mM HEPES, 1 mM EDTA, 0.5 mM DTT, 0.5 mg/mL BSA, 2 mM Trolox, and 50 mM KGlu (pH 7.9). Many DNAs were collapsed as large spots and did not stretch back after relaxing DNAs for 2 s and then stretching DNAs.


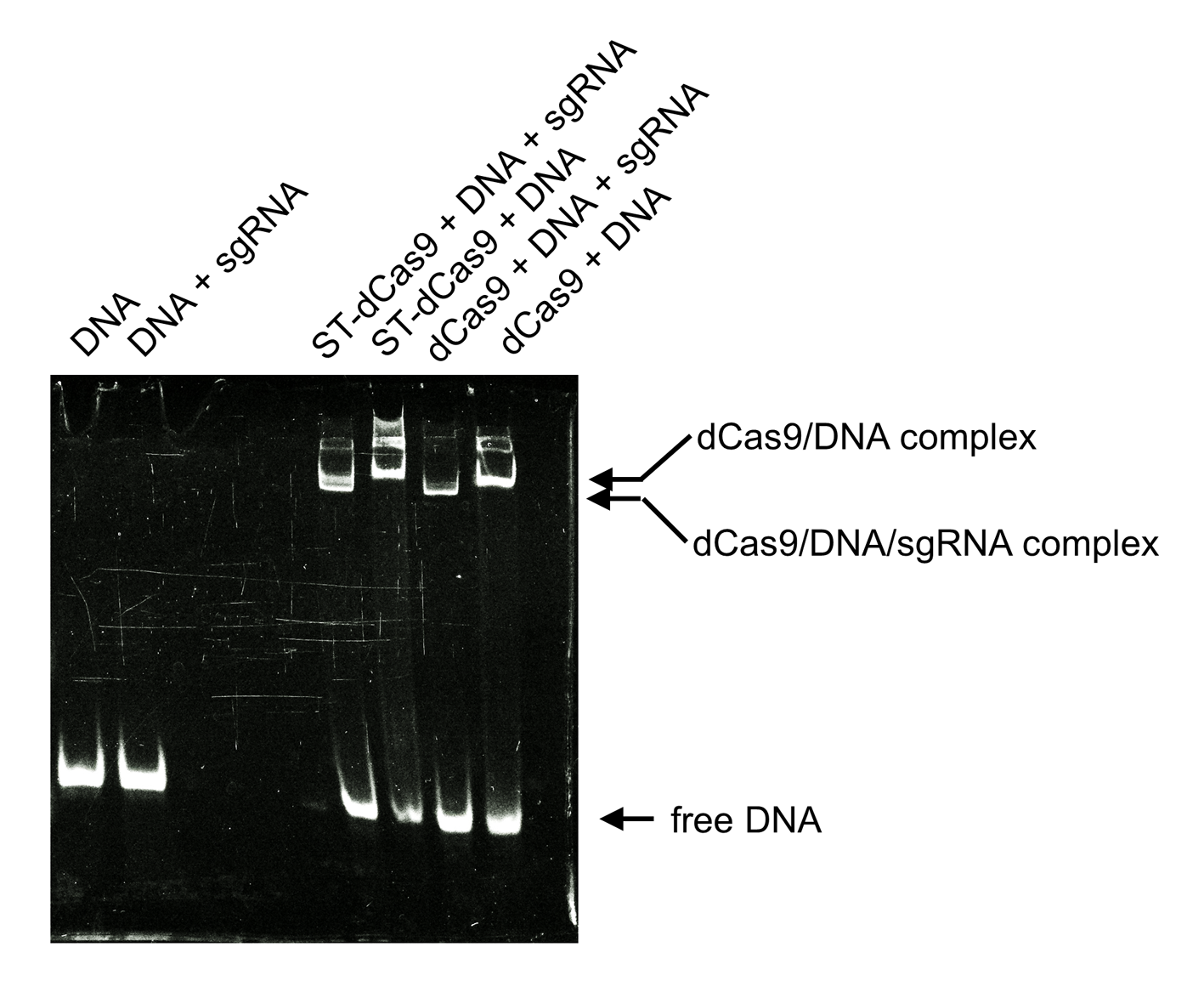


**Fig. S5.** Gel shift assay to confirm the formation of the dCas9, DNA, and sgRNA complex.


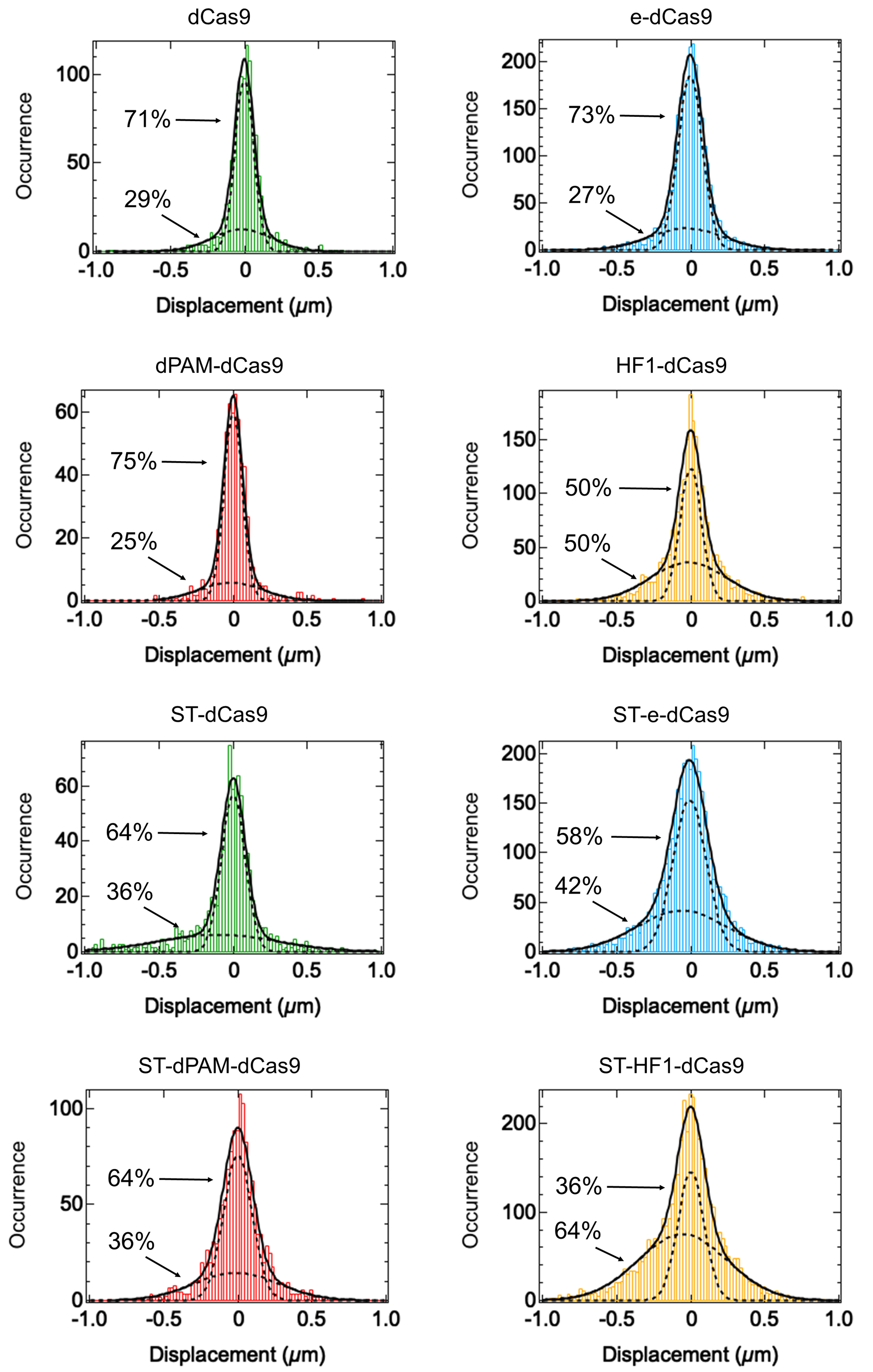


**Fig. S6.** Displacement distribution of dCas9 mutants including hybrids at a time interval of 176 ms in the presence of sgRNA. Black curves are the best-fit curves based on the sum of two Gaussian functions. The dashed curves represent the distributions of each mode.

**Table S1:** Fraction and diffusion coefficient of each sliding mode of the dCas9 mutants in the absence of gRNA

| Cas9 mutant | sliding fraction (%) ^#^ | Averaged *D* (µm^2^/s) ^#^ | | Averaged *D* (µm^2^/s)^$^ |
| --- | --- | --- | --- | --- |
|  |  | **Sliding mode** | **Paused mode** |  |
| dCas9 | 23 ± 6 | 0.07 ± 0.02 | 0.010 ± 0.001 | 0.009 ± 0.003 |
| dPAM-dCas9 | 36 ± 1 | 0.54 ± 0.03 | 0.0224 ± 0.0008 | 0.134 ± 0.007 |
| e-dCas9 | 23 ± 2 | 0.27 ± 0.04 | 0.0120 ± 0.0007 | 0.010 ± 0.007 |
| HF1-dCas9 | 26 ± 4 | 0.09± 0.01 | 0.0124 ± 0.0009 | 0.007 ± 0.002 |
| ST-dCas9 | 29 ± 3 | 0.08 ± 0.01 | 0.0133 ± 0.0008 | 0.011 ± 0.002 |
| ST-dPAM-dCas9 | 38 ± 3 | 0.18 ± 0.02 | 0.012 ± 0.001 | 0.065 ± 0.006 |
| ST-e-dCas9 | 34 ± 2 | 0.14 ± 0.01 | 0.0161 ± 0.0009 | 0.036 ± 0.003 |
| ST-HF1-dCas9 | 42 ± 3 | 0.12 ± 0.01 | 0.013 ± 0.001 | 0.039 ± 0.003 |

The ^#^*D* value of each mode was determined by fitting the displacement distribution. ^$^ Average *D* values were determined using MSD plots of all trajectories.

**Table S2:** Fraction and diffusion coefficient of each sliding mode of the dCas9 mutants in the presence of gRNA

| Cas9 mutant | sliding fraction (%) ^#^ | Averaged *D* (µm^2^/s) ^#^ | | Averaged *D* (µm^2^/s)^$^ |
| --- | --- | --- | --- | --- |
|  |  | **Sliding mode** | **Paused mode** |  |
| dCas9 | 29 ± 4 | 0.11 ± 0.02 | 0.011 ± 0.001 | 0.021 ± 0.005 |
| dPAM-dCas9 | 25 ± 5 | 0.13 ± 0.04 | 0.011 ± 0.001 | 0.018 ± 0.006 |
| e-dCas9 | 27 ± 3 | 0.18 ± 0.02 | 0.020 ± 0.001 | 0.039 ± 0.004 |
| HF1-dCas9 | 50 ± 4 | 0.18 ± 0.01 | 0.015 ± 0.002 | 0.104 ± 0.007 |
| ST-dCas9 | 36 ± 3 | 0.46 ± 0.08 | 0.018 ± 0.002 | 0.17 ± 0.01 |
| ST-dPAM-dCas9 | 36 ± 4 | 0.23 ± 0.03 | 0.026 ± 0.003 | 0.060 ± 0.006 |
| ST-e-dCas9 | 42 ± 3 | 0.26 ± 0.02 | 0.036 ± 0.003 | 0.109 ± 0.005 |
| ST-HF1-dCas9 | 64 ± 3 | 0.26 ± 0.01 | 0.022 ± 0.002 | 0.164 ± 0.005 |

The ^#^*D* value of each mode was determined by fitting the displacement distribution. ^$^ Average *D* values were determined using MSD plots of all trajectories.

**Table S3:** Oligonucleotide sequences used for sgRNA preparation

| 5’-DNA template for transcription | AAACAAGCTAATACGACTCACTATAGGACGCATAAAGATGAGACGCGTTTTAGAGCTATGCTGTTTTGGAAACAAAACAGCATAGCAAGTTAAAATAAGGCTAGTCCGTTATCAACTTGAAAAAGTGGCACCGAGTCGGTGCTTTTTTTGGATC |
| --- | --- |
| 3’-DNA template for transcription | GATCCAAAAAAAGCACCGACTCGGTGCCACTTTTTCAAGTTGATAACGGACTAGCCTTATTTTAACTTGCTATGCTGTTTTGTTTCCAAAACAGCATAGCTCTAAAACGCGTCTCATCTTTATGCGTCCTATAGTGAGTCGTATTAGCTTGTTT |
| sgRNA | GGACGCAUAAAGAUGAGACGCGUUUUAGAGCUAUGCUGUUUUGGAAACAAAACAGCAUAGCAAGUUAAAAUAAGGCUAGUCCGUUAUCAACUUGAAAAAGUGGCACCGAGUCGGUGCUUUUUUUGGAUC |

The underlined sequence denotes the complementary sequence for the target of λDNA
